# Finite-element-method (FEM) model generation of time-resolved 3D echocardiographic geometry data for mitral-valve volumetry

**DOI:** 10.1186/1475-925X-5-17

**Published:** 2006-03-03

**Authors:** Janko F Verhey, Nadia S Nathan, Otto Rienhoff, Ron Kikinis, Fabian Rakebrandt, Michael N D'Ambra

**Affiliations:** 1 MVIP ImagingProducts GmbH, Nörten-Hardenberg, Germany; 2 Department of Medical Informatics, University Hospital Göttingen, Göttingen, Germany; 3 Department of Anesthesiology, Ohio State University, Columbus, Ohio, USA; 4 Department of Anesthesiology, Brigham and Women's Hospital, Boston, USA; 5 Surgical Planning Laboratory, Department of Radiology, Brigham and Women's Hospital, Boston, USA

## Abstract

**Introduction:**

Mitral Valve (MV) 3D structural data can be easily obtained using standard transesophageal echocardiography (TEE) devices but quantitative pre- and intraoperative volume analysis of the MV is presently not feasible in the cardiac operation room (OR). Finite element method (FEM) modelling is necessary to carry out precise and individual volume analysis and in the future will form the basis for simulation of cardiac interventions.

**Method:**

With the present retrospective pilot study we describe a method to transfer MV geometric data to 3D Slicer 2 software, an open-source medical visualization and analysis software package. A newly developed software program (ROIExtract) allowed selection of a region-of-interest (ROI) from the TEE data and data transformation for use in 3D Slicer. FEM models for quantitative volumetric studies were generated.

**Results:**

ROI selection permitted the visualization and calculations required to create a sequence of volume rendered models of the MV allowing time-based visualization of regional deformation. Quantitation of tissue volume, especially important in myxomatous degeneration can be carried out. Rendered volumes are shown in 3D as well as in time-resolved 4D animations.

**Conclusion:**

The visualization of the segmented MV may significantly enhance clinical interpretation. This method provides an infrastructure for the study of image guided assessment of clinical findings and surgical planning. For complete pre- and intraoperative 3D MV FEM analysis, three input elements are necessary: 1. time-gated, reality-based structural information, 2. continuous MV pressure and 3. instantaneous tissue elastance. The present process makes the first of these elements available.

Volume defect analysis is essential to fully understand functional and geometrical dysfunction of but not limited to the valve. 3D Slicer was used for semi-automatic valve border detection and volume-rendering of clinical 3D echocardiographic data. FEM based models were also calculated.

**Method:**

A Philips/HP Sonos 5500 ultrasound device stores volume data as time-resolved 4D volume data sets. Data sets for three subjects were used. Since 3D Slicer does not process time-resolved data sets, we employed a standard movie maker to animate the individual time-based models and visualizations.

Calculation time and model size were minimized.

Pressures were also easily available. We speculate that calculation of instantaneous elastance may be possible using instantaneous pressure values and tissue deformation data derived from the animated FEM.

## Introduction

Mitral valve defects are a common source of severe mitral regurgitation. 3D echocardiography, especially the intraoperative TEE, allows a detailed assessment of the valve leaflets and commissures [1]. It is currently available in most cardiac surgical operating rooms. In some centers, intraoperative 3D echocardiography is used to evaluate geometry and to plan surgical interventions prior to mitral valve remodeling surgery. However, quantitation of mitral valve geometry is limited to rather imprecise measures. Thus, the cardiac surgeon has no sophisticated, immediate, quantitative analysis of the preoperative 3D mitral valve geometry. Intraoperative quantitative analysis of the dynamic geometry of the mitral valve might provide new information upon which to base more precise, patient-specific planning of the surgical intervention. After the operation, assessment of the repair in a precise and quantitative fashion while the valve is fully operational may allow a better understanding of long term outcome predictors.

Because the mitral valve cannot be realistically described by a symmetric mathematical model [2], the modern approach consists of using a FEM mesh which can approximate the cardiac geometry [3-5]. A very good overview on this theme is given by J. Mackele [6].

Initial attempts at FEM in the heart have been carried out with 3D segmentation and tracking using sophisticated and expensive cardiac MRI [7-9]. MRI is impractical in the cardiac surgical operating room and is complicated by the fact that the mitral valve and the papillary muscles are active materials behaving differently during systole and diastole. An ideal model would provide material properties specific to each patient as first mentioned by McCulloch [10], but until now patient-specific modeling in the operating room has not been possible.

FEM modeling of 3D intraoperative echo data provides an excellent tool for incorporating material properties, volumetric data and boundary pressures to more accurately record, and then to simulate mitral valve dynamic performance. Accurate simulation will be the foundation of surgical planning. The limitation until now in applying FEM intraoperatively has been the technical complexity of this technique. The purpose of this study is to take the first step towards introducing FEM for mitral valve volumetry into the operating room environment. The goal is to facilitate transfer of geometric data from the 3D ultrasound data set into FEM models and there from to further visualization and simulation processing.

## Materials and methods

**Figure 1:**
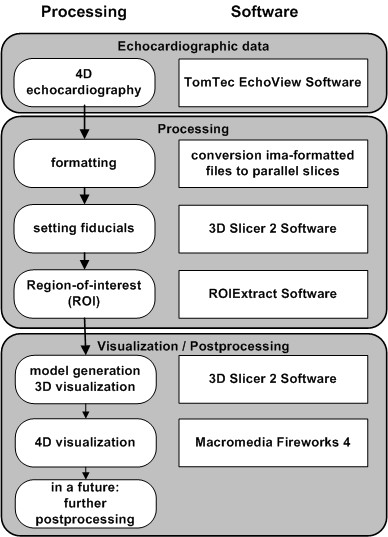
**Strategy to process the data sets**. The processing diagram shows the workflow used in the study beginning with the echocardiographic data acquisition and ending with the geometry model generation with 3D Slicer 2 software package.

**Figure 2:**
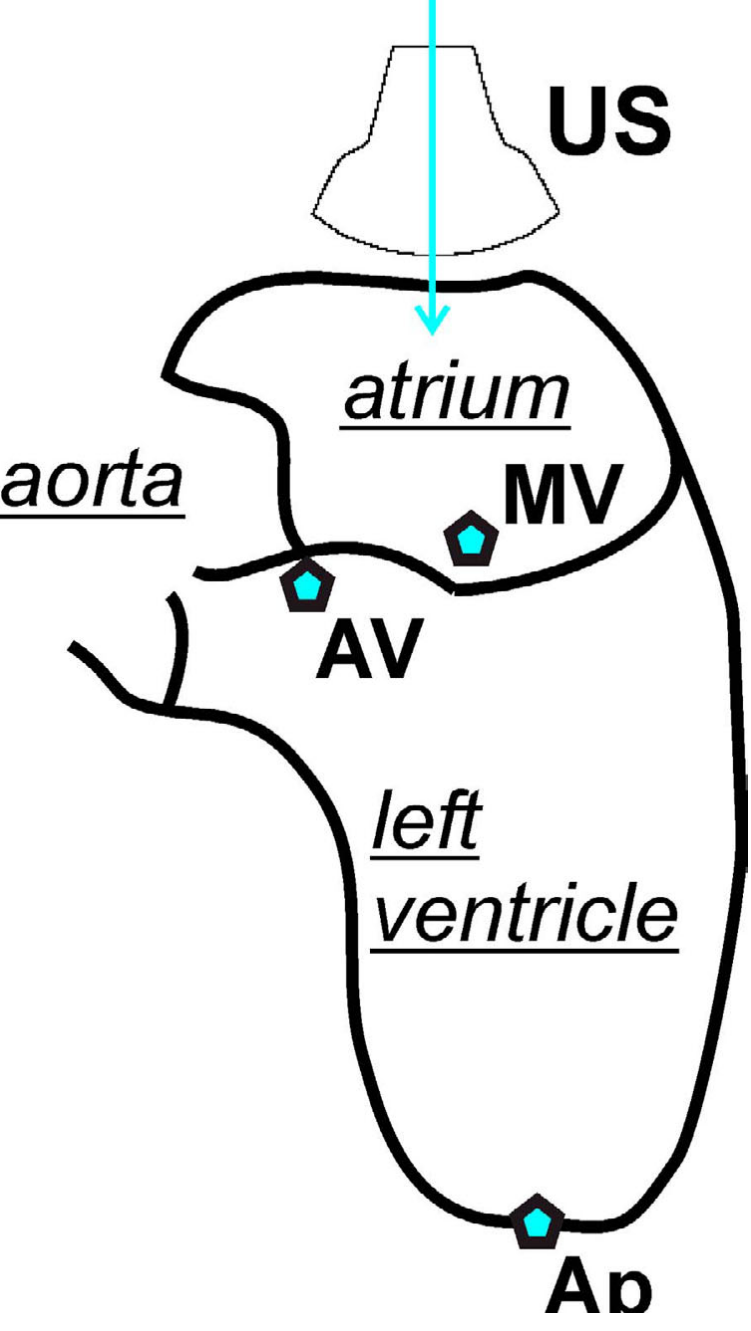
**Section through left atrium and ventricle shown schematically**. US indicates the position of the TEE transducer and the beam direction. AV is aortic valve, MV is mitral valve and Ap is apex.

The present retrospective study is based on a workflow which is shown in detail in Figure 1. Mitral valve images from clinical TEE data sets were obtained in three patients using a Philips Sonos 5500 echocardiographic device. After induction of general anesthesia and airway protection, the esophagus was intubated using an omniplane TEE transducer (see Figure 2).

3D TEE data sets of the mitral valve structures, especially the leaflets, were obtained using the automated Philips acquisition protocol at 3°, 10° and 15° increments respectively (see Table 1).

**Table 1:** General data set parameters

reference number in the article	number of time steps	resolution time [s]	increment	spatial resolution		
				
				x [mm]	y [mm]	z [mm]
1	24	0.0331	3°	0.3665	0.5863	0.3665
2	20	0.0334	15°	0.8143	1.3029	0.8143
3	36	0.0333	10°	0.3665	0.5863	0.3665

General data set parameters for the patient data sets used in this retrospective study taken from the original header of the Echo-View data sets.

Images were gated for both beat-to-beat variability and respiratory motion. All images were stripped of patient identifiers. The time dependent 3D data sets acquired from the Philips Sonos were arranged and stored on a Windows XP based standard PC with TomTec Echo-View^© ^software [11] in a propeller-like geometry, in which the images intersect along the scanning axis [12]. For mitral valve geometry reconstruction, a newly developed software program preprocessed the data by reformatting the data into parallel slices.

**Figure 3:**
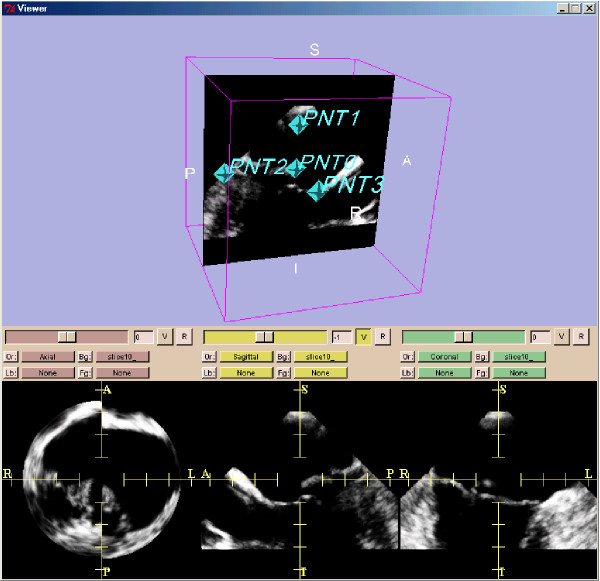
**Ultrasound data of after preprocessing**. The ultrasound data are shown from patient 1. Shown is a standard visualization of one time step during the heart cycle with 3D Slicer 2 software. The three planes for each spatial direction are shown in the lower three frames. In the frame above one plane is shown together with the set of four fiducials. These four points are defined for all time steps.

**Figure 4:**
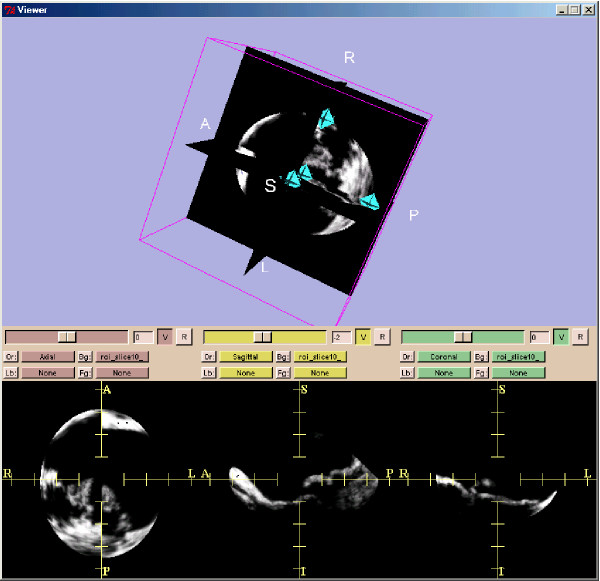
**Ultrasound data after applying the elliptical region-of-interest (ROI)**. Ultrasound data of the same patient as in Figure 2 after applying the elliptical region-of-interest (ROI) defined by the four fiducials (above frame). The three planes for each spatial direction are shown in the lower three frames.

Further processing was carried out with the 3D Slicer software package [13] which is able to process parallel slice data sets. 3D Slicer was run on a SunBlade 2000 with Sun Solaris 5.9 operating system which imports, analyzes, reports and archives the time-resolved 3D-ultrasound data sets. 3D Slicer also runs on standard personal computer operating systems such as Linux and OS X. The first landmark for the region-of-interest (ROI) was set in the middle of the mitral valve at the level of its annulus. Three additional landmarks to mark the ends of the three principal axes of the elliptic ROI were placed in the tissue surrounding the mitral valve (Figure 3 and Figure 4). The landmarks were set inside the tissue in a distance of some five millimeters away from the mitral valve in order to avoid detrimental border effects in the creation of the model. Care was taken to avoid having the mitral tissue cross the borders of the ROI at any point in the cardiac cycle. Using the landmarks, another software module developed for this project allows selection of an elliptic ROI. With this landmarking procedure, a time-resolved mitral valve geometric analysis with a FEM model for each time point in the cardiac cycle was obtained (see Results) by the 3D Slicer.

Time-resolved images of the rendered mitral valve geometry resulting from the 3D Slicer were transferred to a standard movie maker (Dreamweaver Fireworks 4) [14] running on a Fujitsu-Siemens laptop with Windows XP operation system in order to obtain animations for visualization purposes.

The quantification of distances and volumes can be carried out with 3D Slicer.

The time required for each step in this process was recorded for each patient data set (see Table 2).

**Table 2:** Data acquisition and processing times.

data set	ultrasound acquisition [s]	pre-processing: reformatting, ROI-application [s]	FEM modelling with 3D Slicer [s]	post-processing moviemaker [s]	total processing time [s]
1	725	440	2860	355	4380
2	605	360	2610	240	3815
3	660	470	4310	390	5830
Mean [s]	663	423	3260	391	4675

On average each processing took 78 minutes taking into acount the large difference in the number of time steps taken between patient 2 and 3, respectively.

## Results

The Philips Sonos 5500 ultrasound system required around 11 minutes acquisition time per patient. The application of the reformatting software, the ROI algorithm with manual placement of the necessary landmarks took approximately 7 minutes per patient. In three patient data sets, conversion from TomTec Echo-View data to the 3D Slicer FEM model was carried out in 2 minutes for each time step of the heart cycle. On average, the processing time took 54 minutes per patient. Image acquisition and movie production on the above computer was 30 seconds per sequence or approximately 6 minutes per patient. Total time for the procedure was approximately 78 minutes per patient (see Table 2).

**Figure 5:**
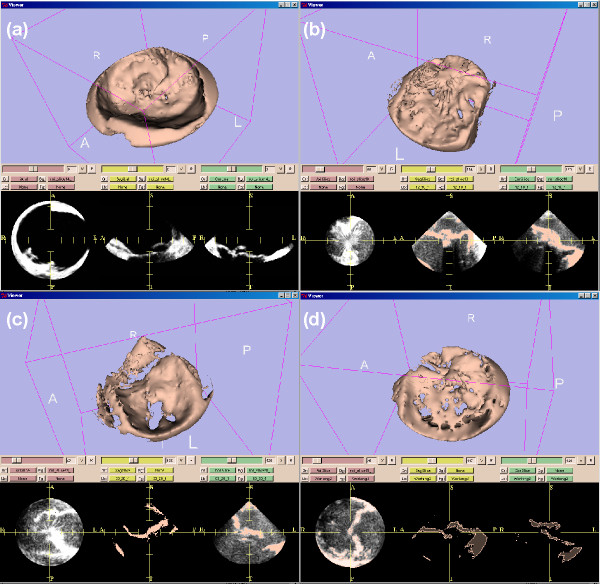
**3D FEM models at systole for the three data sets**. (a) patient 1, (b) patient 2, (c) and (d) patient 3 at two different time steps in the cardiac cycle. Here a smoothing factor [13] of 20 and a decimation factor [13] of 20 were applied in order to obtain FEM models with a reasonable number of FEM triangles for further processing. 3D FEM model of the same data set as in Figures 3 and 4. The three planes (some with level sets) for each spatial direction show the cutting planes through the model. No smoothing or decimation was applied.

**Figure 6:**
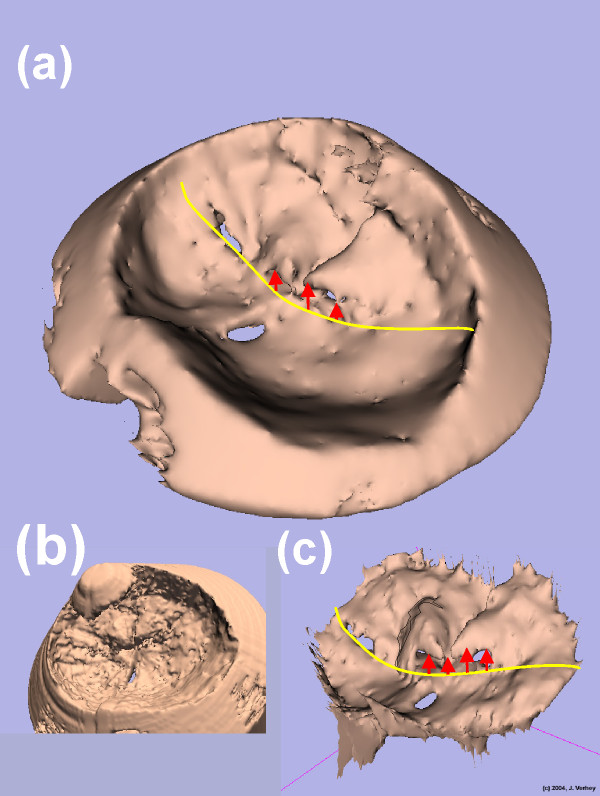
**Models from a patient using different parameters and techniques**. In this figure FEM models from patient 1 are shown in different manners. (a) is the smoothed and decimated model shown in Figure 5 (a). In addition the yellow line indicates the leaflets border. The red arrows indicate the prolapse of the mitral valve leaflet. (b) is a model where no ROI was applied so the full (and unnecessary) ultrasound cone is visible. (c) shows a model based on a very time consuming manual segmentation.

**Figure 7:**
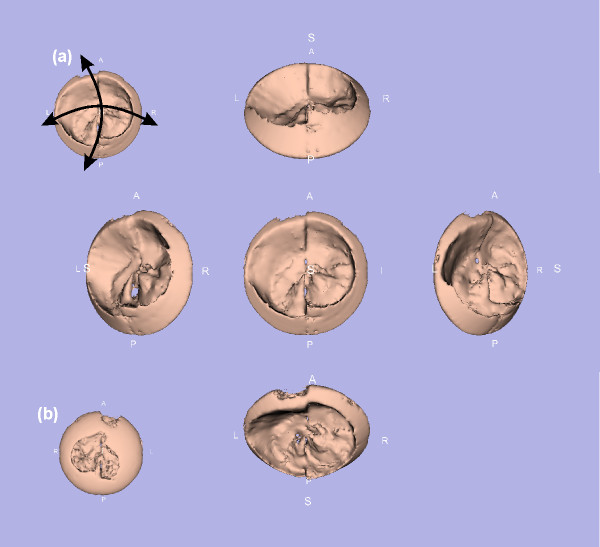
**Rotating 3D model to get the best point of view**. This figure illustrates the choice of the best point of view from a single model. (a) The 3D model in the viewer window can be moved and rotated in order to get the best view to the morphological findings. (b) shows the back view.

Figure 5 shows the FEM models at systole for all of the data sets and shows the FEM model at systole for data set 1 after model creation with 3D Slicer. No smoothing or decimation [13] was used to create this model. Figure 6 shows three different model stages ((c) manually segmented, (b) without ROI and (a) with ROI application) for one of the patient with a mitral valve prolapse. As a result, detailed structures from the ultrasound data were modeled and model files were generated especially the models with manual segmentation and with no reduction by application of the ROI algorithm. The ROI minimizes the number of FEM triangles, which is necessary for further processing. All of the models were calculated with a smoothing and a decimation factor of 20 each [13]. As a result the number of FEM triangles significantly decreased to 10 percent of the model. Figure 7 shows one FEM model taken from different points of view processed for visualization (below) in order to show the manoeuvrability with the 3D Slicer software. With a standard movie maker [14], the view of models through the heart cycle was animated [see Additional file 1]. Similar visualizations are taken from each of the data sets which are not shown separately here. These figures demonstrate that a quantification of movement during the heart cycle is directly possible using calculated continuous mitral valve FEM models.

## Discussion

The primary intention of this study was to demonstrate the feasibility of transporting individual patient's mitral valve geometry data into a FEM model. Standard PC/workstation computer technology was utilized to accomplish the transfer from a common TEE-machine (Philips Sonos 5500).

The data acquisition software were commercially available TomTec Echo View^© ^package and open-source 3D Slicer 2 software, plus recently developed components for data set reformatting. Accomplishing this transfer forms a foundation for quantitative approaches to intraoperative surgical planning and outcomes assessment in valvular reconstructive surgery.

This method would also be applicable to next generation "live 3D" systems, 3D ultrasound data sets obtained from matrix array transducers.

The total time required for acquisition to a completed FEM model was approximately 1hr 15 minutes and can be accomplished during the time period when the patient is being prepared for cardiopulmonary bypass (generally 1 to 1.5 hr). Thus the feasibility in terms of duration is demonstrated. The total time of processing could be surely decreased in the future if a single computer platform would be used. All used programs run e.g. on Windows XP systems. For this feasibility study we used a combination of three platforms just for our convenience and in order not to disturb the intervention itself.

In terms of procedure accuracy, reproducibility and duration, the primary limitation is the dependence of the newly developed software on manual entries of the four landmarks. Manual entries must be done for multiple frames within the TEE data sets and as a consequence the method involves some degree of inter- and intraobserver variability which is a general problem for ultrasonic imaging. The reproducibility of these measurements will require further study.

In Figure 3 the left of the three orthogonal planes at the bottom demonstrates an uncontinuous heart wall. This shows that the quality of the calibration of the transducer might be enhanced from the medical point of view. These data sets basically were taken during an intervention and not in an in-vitro experiment. From the technical point of view this doesn't limit the usability of the method described in the study.

A limitation of the present study is that it is focused only on the deployment of the transfer method. The tool for modelling is not within the scope of this article. Nevertheless, quantification the method makes it immediately available for mitral valve leaflet motion, shape and volume analysis. Instantaneous analysis has a number of potential applications for mitral valve function assessment and surgical planning. These applications require both comprehensive automated valve leaflet motion analysis as well as quantification of dynamic mechanical properties through a biomechanical FEM of the mitral valve region.

The scope of this study was to produce a prototype in which the feasibility of the method could be assessed. In a fully operational system, we postulate clinical applications such as enhanced/automated leaflet motion abnormality detection, assessment of regional relaxation which encompasses the entire ventricle, assessment and guidance of ventricular remodeling operations, and serial assessment of recovery of regional wall function post myocardial stunning

FEM meshes have been used for approximately 30 years [6,15] in the analysis of many anatomical structures and organs such as major vessels [16,17], heart valves [18] and ventricles [4,19], lung [20], corneoscleral shell [21], plastic and reconstructive craniofacial surgery [22] and the femur [23]. A FEM model can be created to determine the deformation of the mitral valve loaded by intraventricular pressure. Steady-state fluid dynamics and structural analyses can be carried out using commercial codes based on FEM [24]. At a sequence of time-steps in the cardiac cycle, the valve leaflet can be considered to be a quasi-incompressible, transversely isotropic hyperelastic material based on the analysis of Feng [25]. Until now, biomechanical cardiac FEM models have been based on a simplified ellipsoidal and cylindrical geometries [25] or asymmetric modelling [2]. A FEM created in this way is not patient-specific and does not accurately represent precise regional deformations in a cardiac structure such as the mitral valve loaded by intraventricular pressure. The method described here will allow patient specificity and the precise representation of leaflet deformation. Precise deformation data is required to allow elastance determinations critical for simulation of the response of the tissue to virtual interventions.

## Conclusion

For complete intraoperative 3D mitral valve finite element analysis, three input elements are necessary: 1. time-gated, reality-based structural information, 2. continuous left ventricle pressure, and 3. instantaneous tissue elastance. The first of these elements is now available using the methods presented herein. The later two parameters will be required for further robust modeling and analysis. FEM analysis has not been feasible for mitral valve in the intraoperative setting. The major roadblock was the complexity and the practicality of transfer of structural 3D data to a FEM analysis program. This study describes a method to rapidly transfer 3D structural data from the TEE device into a FEM model which can be loaded easily by standard FEM analysis programs. Once measured pressure and calculated elastance are added to the model, near real-time dynamic stress-strain information in the operating room will be achievable.

## Authors' contributions

JFV being the coordinator did the developing, the technical part implementing the workflow to create the FEM models and the FEM modelling with 3D Slicer itself. OR organized and provided the technical equipment to calculate the mitral valve FEM models. RK advised on the use of 3D Slicer software package for 4D applications. FR did the development and programming of the additional software components. MNDA and NSN who were the germ cell of the idea for this kind of modelling acquired the data and medical part, especially in advisory capacity for the setting of the fiducials. All authors read and approved the final manuscript.

## Supplementary Material

Additional File 1 Animated visualization of the 3D geometrical FEM models. Shown are the time steps during the heart cycle (patient 1) as animated GIF. Click here for file
