# Supplementary figures and images for: Finite-element-method (FEM) model generation of time-resolved 3D echocardiographic geometry data for mitral-valve volumetry
Source: Biomed Eng Online. 2006 Mar 3;5:17. doi: 10.1186/1475-925X-5-17 (PMC1421418; doi:10.1186/1475-925X-5-17)

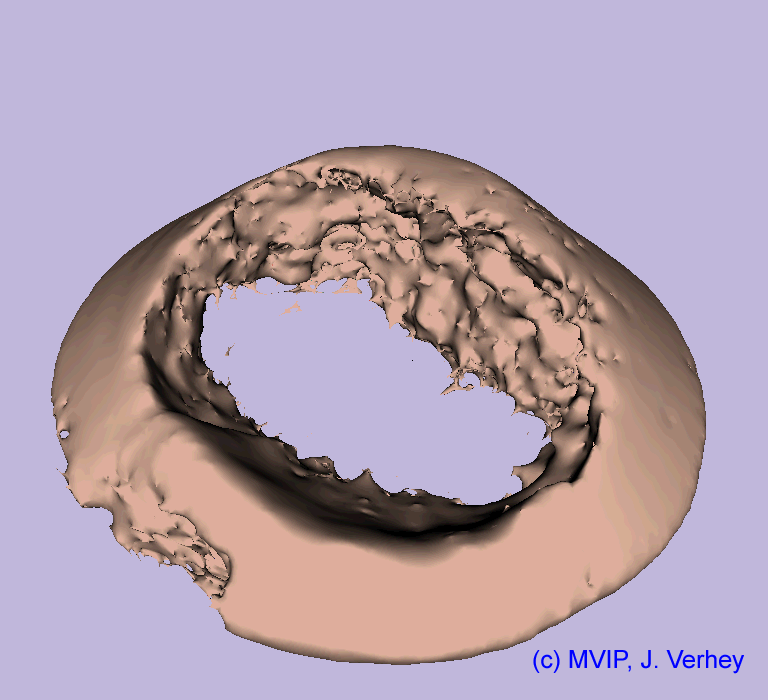

Supplement: Additional File 1 — Animated visualization of the 3D geometrical FEM models. Shown are the time steps during the heart cycle (patient 1) as animated GIF. [file 1475-925X-5-17-S1.gif]
